# Contribution of *Saccharomyces* and Non-*Saccharomyces* Yeasts on the Volatile and Phenolic Profiles of Rosehip Mead

**DOI:** 10.3390/antiox12071457

**Published:** 2023-07-19

**Authors:** Alexandra-Costina Avîrvarei, Carmen Rodica Pop, Elena Mudura, Floricuța Ranga, Simona-Codruța Hegheș, Emese Gal, Haifeng Zhao, Anca Corina Fărcaș, Maria Simona Chiș, Teodora Emilia Coldea

**Affiliations:** 1 Department of Food Engineering, Faculty of Food Science and Technology, University of Agricultural Sciences and Veterinary Medicine Cluj-Napoca, 400372 Cluj-Napoca, Romania; costina.avirvarei@gmail.com (A.-C.A.); elena.mudura@usamvcluj.ro (E.M.); simona.chis@usamvcluj.ro (M.S.C.); 2 Department of Food Science, University of Agricultural Sciences and Veterinary Medicine, 400372 Cluj-Napoca, Romania; carmen-rodica.pop@usamvcluj.ro (C.R.P.); floricuta.ranga@usamvcluj.ro (F.R.); anca.farcas@usamvcluj.ro (A.C.F.); 3 Department of Drug Analysis, Faculty of Pharmacy, “Iuliu Hatieganu” University of Medicine and Pharmacy 6, Louis Pasteur Cluj-Napoca, 400349 Cluj-Napoca, Romania; cmaier@umfcluj.ro; 4 Faculty of Chemistry and Chemical Engineering, Babeș-Bolyai University, 11 Arany Janos Street, 400028 Cluj-Napoca, Romania; emese@chem.ubbcluj.ro; 5 School of Food Science and Engineering, South China University of Technology, Guangzhou 510640, China; hfzhao@scut.edu.cn; 6 Research Institute for Food Nutrition and Human Health, Guangzhou 510640, China

**Keywords:** rosehip, mead, yeast, volatile profile, phenolic profile, antioxidant activity

## Abstract

The resurgence of mead, a honey-based fermented beverage, is attributed to the increasing consumption of fermented foods and beverages, driven by its distinct flavors and perceived health benefits. This study investigates the influence of different yeast strains, namely *Saccharomyces cerevisiae* var. *bayanus*, and *Torulaspora delbrueckii*, on the volatile and phenolic compounds of these beverages. Analytical techniques, including HPLC-DAD and GS/MS, were employed to analyze the chemical composition of the beverages. ANOVA analysis of variance was conducted to assess differences in the volatile and phenolic compounds. The findings reveal that yeast selection significantly impacts the chemical profiles of the beverages. *Saccharomyces cerevisiae* fermentation preserves rosehip-specific flavonoids and phenolic acids. Sequential fermentation with *Torulaspora delbrueckii* demonstrated proficiency in generating esters, contributing to fruity and floral aromas in the beverages. This study investigates the importance of yeast selection in shaping the chemical composition of rosehip mead, providing insights into the distinct characteristics conferred by different yeast strains. By optimizing yeast selection and fermentation techniques, the overall quality and diversity of these beverages can be enhanced.

## 1. Introduction

Rosehip (*Rosa canina* L.) is a pseudofruit derived from the rose plant, scientifically recognized for its abundance of polyphenols, vitamin C, and a diverse array of bioactive compounds [1,2,3]. Most often found as a wild shrub, *Rosa canina* L. is now gaining popularity as an industrial crop [4]. It is indigenous to Europe, Northwest Africa, and Western Asia, and holds a prominent position in traditional medicine for its profound prophylactic and therapeutic effects against a range of ailments. Scientific evidence supports the potent anti-inflammatory [5], antioxidant [6], and antiobesogenic activities [7].

Mead is defined as an alcoholic beverage obtained through the fermentation of a diluted honey solution by yeast, with an ethanol content ranging from 4 to 14% (*v*/*v*) at 20 °C [8], with the possibility of incorporating additional ingredients such as fruits, herbs, or spices [9]. The manufacturing process of mead typically involves fermenting a diluted honey solution using yeast, resulting in the production of an alcoholic beverage [10]. Fermentation, a pivotal stage, significantly affects the chemical composition and quality of mead, with outcomes influenced by various factors including fermentation conditions, yeast strains, and technological treatments [11]. The presence of valuable compounds, such as sugars, organic acids, vitamins, minerals, and antioxidants, contribute to the nutritional and sensory properties of meads [12].

Mead exhibits promising functionalities owing to its intricate chemical composition. Notably, meads have demonstrated noteworthy antioxidant properties attributed to the presence of polyphenolic compounds and other bioactive constituents derived from added ingredients such as rosehips [13]. These constituents, encompassing proanthocyanidins, anthocyanins, flavonoids, and phenolic acids, contribute to the antioxidant, anti-inflammatory, and antiviral activities associated with mead consumption [14]. Moreover, research efforts have focused on optimizing mead production parameters, particularly investigating manufacturing techniques and exploring the impacts of mixed fermentations utilizing diverse yeast strains on the composition and antioxidant properties of mead [15].

*Saccharomyces cerevisiae* has played a crucial role in the production of alcoholic beverages with an extensive history deeply rooted in the area of fermentation, spanning over eight millennia. Its fermentation process yields a diverse range of flavor compounds, including alcohols, aldehydes, organic acids, esters, organic sulfides, and carbonyl compounds, which greatly influence the sensory characteristics and overall quality of fermented foods and beverages [16,17]. This intricate balance of low-molecular-weight compounds serves as a unique organoleptic signature, distinguishing specific products and brands [18].

In winemaking, *Saccharomyces bayanus*, a yeast species closely related to other *Saccharomyces* strains, demonstrates distinct oenological properties and the ability to synthesize volatile compounds [19]. Notably, *S. bayanus* exhibits heightened flavor intensity, producing significant quantities of compounds such as 2-phenylethanol, ethyl lactate, and acetate esters [20]. By leveraging the contributions of *S. bayanus*, winemakers can enhance sensory attributes and augment aromatic complexity, particularly through the development of floral, fruity, and spicy notes associated with 2-phenylethanol and its esters [21].

Another noteworthy yeast species, *Torulaspora delbrueckii*, has garnered attention in winemaking due to its potential for elevating wine quality. *T. delbrueckii* displays desirable characteristics such as low acetic acid production, reduced ethanol concentrations, increased glycerol content, and enhanced mouthfeel properties [22]. Moreover, *T. delbrueckii* positively influences wine aromatics by promoting the formation of fruity esters, thiols, and terpenes while mitigating higher alcohol levels [23].

Traditionally, fermentation has relied on monocultures of yeast strains to ensure consistent aroma profiles and a relatively high ethyl alcohol content. However, *Saccharomyces* species or alternative *Saccharomyces* strains have been exploder in the last decade. This pursuit aims to capture characteristic flavors, higher concentrations of desirable esters, and heightened aroma complexity [23,24]. Spontaneous fermentation, initiated by non-*Saccharomyces* yeasts such as *T. delbrueckii*, followed by the contribution of *Saccharomyces* cultures, has emerged as a viable approach to achieve these desired sensory outcomes [25].

Despite its historical significance and prevalence in Eastern European countries, mead currently lags behind other alcoholic beverages in terms of popularity, underscoring the necessity for further scientific exploration of its properties and potential functionalities [26]. According to a market survey published in 2022, the mead key consumer countries are the United States, Canada, China, Germany, and the United Kingdom; with global year-over-year growth of 6.7%, the mead popularity is continuously increasing [27].

China ranks as the first worldwide honey producer, followed by the European Union, with Romania, Spain, Hungary, Germany, Italy, Greece, France, and Poland, as the most representative countries in this sector [28]. According to Honey Market Presentation Expert Group for Agricultural Markets (2023), the imported honey price increased, ranging from 1.58 Euro/kg (in China) to 4.27 Euro/kg (in the U.K.), making the production of mead questionable in terms of economic sustainability. Still, Poland, Estonia, and Romania are the top three honey exports EU state members to the U.K., each with 1000, 867, and 514 tons, respectively.

National supporting programs for the apiculture sector applied in the EU state members help to increase the production of honey and honeybee products and indirectly offer benefits for agriculture as well. This way, new opportunities may arise for the local producers, such as producing sustainable value-added honeybee-based products. Moreover, the rosehip, presently an underutilized and sustainably grown fruit, also possesses great potential to generate value-added potentially functional products.

Our first objective was to develop a rosehip mead based on local ingredients using *Saccharomyces* and non-*Saccharomyces* yeast strains as single strain and co-fermented experimental variants, respectively. Second, we investigated the compositional analysis of rosehip mead in terms of volatile and phenolic compounds, amino acids, organic acids, and glucides. This investigation contributes to the broader understanding of yeast-driven fermentation and highlights the potential of specific yeast strains in optimizing the chemical attributes of rosehip mead.

## 2. Materials and Methods

This study utilized multifloral honey sourced from a local beekeeper located in close proximity to Piatra Neamt (46°56′41.9″ N, 26°22′17.4″ E) in Northeast Romania. The honey, derived from a combination of acacia (*Robinia pseudoacacia*), linden (*Tilia* spp.), and rape (*Brassica napus*) flowers, exhibited a Brix value of 78.3, indicating its high sugar content.

Fully ripened *Rosa canina* L. fruits, commonly known as rosehips, were harvested from the same region in October 2022. The quality of the fruits was assessed using a DR 201-95 refractometer (Kruss, Hamburg, Germany), which determined a Brix value of 31.7 °Brix. To prepare the rosehip component for subsequent stages of mead production, the fruits were crushed and added to the wort in a proportion of 1:6 (*w*/*v*).

For the mead production process, three yeast strains were employed: *S. cerevisiae* (var. *bayanus*) (Lalvin DV10TM, Lallemand, Montreal, QC, Canada) (V1), *S. cerevisiae* (var. *bayanus*) (Lalvin EC 1118^®^, Lallemand, Canada) (V2), and *T. delbrueckii* (BiovidaTM TD291, Lallemand, Canada) (V3 and V4).

The multifloral honey utilized in this study underwent a purification procedure to ensure its clarity and purity. A 15 g:100 mL ratio of honey to natural water was employed, followed by centrifugation at 10,000× *g* for 10 min to eliminate any impurities and achieve optimal clarity and purity. Additionally, the diluted honey underwent a thermic treatment at 80 °C to mitigate the risk of microbial contamination. To assess the initial composition of the honey wort prior to fermentation, its Brix value was determined using a refractometer (DR 201-95, Kruss, Germany), resulting in a measurement of 10 °Brix. The total acidity of the honey wort was quantified utilizing standard analytical methods, yielding a value of 2.68 g/L tartaric acid. Experimental standards were maintained; all procedures conducted in triplicate to ensure precision and reproducibility.

The reagents used in this study included HPLC-grade acetonitrile (Merck, Darmstadt, Germany), ultrapure water purified with the Direct-Q UV system (Millipore, Burlington, MA, USA), chlorogenic and gallic acid standards (>98% HPLC), rutin, and catechin (>99% HPLC) sourced from Sigma (St. Louis, MO, USA), glucose and fructose standards (purity 99%), oxalic, citric, malic, succinic, lactic, acetic acids, sorbitol, and ethanol alcohols (purity > 99%) obtained from Sigma-Aldrich (Taufkirchen, Germany), and 0.5 M sulfuric acid (Chempur, Piekary Śląskie, Poland). The water used in the experiments was also ultrapure and purified using the Direct-Q UV system from Millipore (USA).

### 2.1. Experimental Design

**Figure 1:**
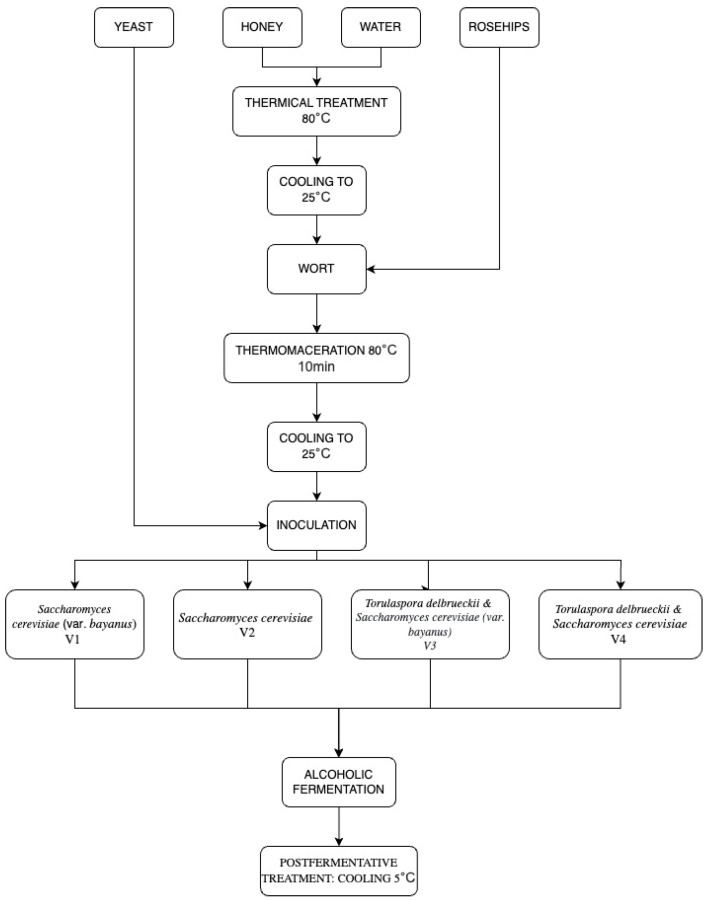
Mead production experimental design and process flow.

The production of mead (Figure 1) commenced with the preparation of wort. The honey wort was subjected to thermal maceration at 80 °C for 10 min, using a rosehip-to-honey wort ratio of 1:6 (*w*/*v*). Following the thermal treatment, the mixture was cooled to 30 °C and inoculated with various yeast species.

The fermentation process involved both single-strain fermentation and sequential fermentations. The single-strain fermentation included *S. cerevisiae* (var. *bayanus*) (V1) and *S. cerevisiae* (V2). For the sequential fermentation, the initial inoculation consisted of *T. delbrueckii*, followed by a second inoculation after 24 h with *S. cerevisiae* (var. *bayanus*) (V3) and *S. cerevisiae* (V4).

Preceding each experiment, a 25 g starter culture was activated by inoculating it with 250 mL of medium at 30 °C. After a cooling period of 15 min, the inoculum was introduced at a dosage of 24 g/L of dry yeast, containing an initial population of 5 × 10^6^ viable cells/mL, adhering to established technical guidelines. To prevent thermal shock and facilitate the acclimation of the culture to the lower fermentation temperature, constant mixing was employed by combining the inoculum with an equal volume of must. Fermentations were conducted in sterile 500 mL flasks, filled to 2/3 of their volume, and maintained at a stable temperature of 5 °C without agitation. The progress of fermentation was monitored throughout the experimental duration, which spanned a period of 31 days. Upon completion of alcoholic fermentation, the mead was decanted, collected, and promptly stored at 5 °C for subsequent analysis and evaluation.

### 2.2. Total Phenolic Content and Antioxidant Activity

TPC was determined according to [29] with some modifications by the spectrophotometric Folin–Ciocalteu method with absorbance in the Vis domain at a wavelength λ of 725 nm. TPC was expressed in relation to a calibration curve with Gallic acid of different concentrations: 1.25 mg/mL, 1 mg/mL, 0.75 mg/mL, 0.50 mg/mL, and 0.25 mg/mL. To plot the calibration curve, the absorbances were read according to Gallic acid concentrations. The calibration curve was: y = 0.715517x + 0.000000, having R2 = 0.99993.

In a 16 mL calibration flask, 0.1 mL of sample and 0.5 mL of FC reagent were added. After shaking the mixture for 4 min, 1.5 mL of sodium carbonate solution (7.5%) was added. Water was used to bring the solution to volume (1.9 mL). The samples were left in the dark for 2 h, and then the absorbance was measured at a wavelength λ of 725 nm with a UV/visible spectrophotometer Schimadzu 1900 (Shimadzu Corporation, Kyoto, Japan). The total quantity of polyphenols was expressed in mg Gallic acid equivalents (GAE)/100 mL product.

The antioxidant activity was determined by using the radical DPPH (2,2-diphenyl-1-picrylhydrazyl) scavenging capacity assay as described by [30].

The samples’ free radical scavenging activity was assessed in comparison to the effects of standard solutions of ethanol Trolox (6-hydroxy-2,5,7,8-tetramethylchroman-2-carboxylic acid) (25–800 µM/mL). The obtained calibration curve had the following equation: y = −0.00104911x + 0.929809 and R2 = 0.99447. The samples (0.15 mL) were mixed with DPPH solution (2.85 mL), kept in the dark at ambient temperature, and the absorbance of the mixtures was recorded at 515 nm after exactly 60 min against methanol as blank. The antioxidant activity was calculated using the Trolox calibration curve.

### 2.3. Volatile Compounds by GS/MS Analysis

Gas chromatographic analysis of volatile compounds was performed using a GC-MS Shimadzu QP 2010 PLUS Mass Spectrometer coupled with Gas Chromatograph (Shimadzu equipped with an AOC-20i+s injector and a highly polar polyethylene glycol stationary phase MS capillary column (30 m × 0.25 mm, 0.25 µm film thickness, ZB-Wax Phenomenex) [31]. The temperature of the injector and the MS transfer line was set to 220 °C. The starting oven temperature was 40 °C, for 5 min, then programmed to rise from 40 °C to 220 °C, at 4 °C/min, and held at this temperature for 15 min. The carrier gas was helium at a constant flow rate of 0.8 mL/min. The injection volume was 1 µL, made in split mode (20:1) at 220 °C. The mass spectral detector was a quadrupole mass spectrometer operated in the full spectral acquisition mode. The mass spectrometer was equipped with an electron ionization source, which was operated with an electron energy of 70 eV.

### 2.4. Odour Activity Values

The odor activity value (OAV) is a measure of importance of a specific compound to the odor of a sample. The OAVs were calculated for the most abundant volatile compound found in analyzed meads. OAVs were obtained by dividing the concentration of the compound by its odor threshold found in the literature [12,15,32,33,34,35].

### 2.5. Phenolic Compounds by HPLC-DAD ESI+

According to [36], the phenolic characteristics were performed using HPLC-DAD ESI+. The experiments were filtrated through the Chromafil Xtra nylon filter of 0.45 µm and 20 μL and further injected into the HPLC system. The HPLC Agilent 1200 system was equipped with a quaternary pump, solvent degasser, autosampler, and UV-Vis detector with a photodiode (DAD), coupled with a single quadrupole mass detector (MS) Agilent model 6110 (Agilent Technologies, CA, USA). Compound separation was performed on a Kinetex XB C18 column, size 4.6 × 150 mm, with 5 μm particles (Phenomenex, Torrance, CA, USA), using the mobile phases (A) water + 0.1% acetic acid and (B) acetonitrile + 0.1% acetic acid in the gradient below, for 30 min, at a temperature of 250 °C, at a flow rate of 0.5 mL/min. Gradient (expressed as % B): 0 min, 5% B; 0–2 min, 5% B; 2–18 min, 5–40% B; 18–20 min, 40–90% B; 20–24 min, 90% B; 24–25 min, 90–5% B; 25–30 min, and 5% B. Spectral values were recorded in the 200–600 nm range for all peaks. Chromatograms were recorded at wavelengths λ = 280 and 340 nm. For the MS the ESI positive full scan ionization mode was used under the following working conditions: capillary voltage 3000 V, temperature 3500 C, nitrogen flow 7 l/min, and m/z 120–1200. All experiments were conducted in triplicate and the reported data are mean values. The obtained concentrations of the most abundant volatile compounds were divided by the odor threshold values derived from the information available in the literature. Concentration data were translated into odor activity values (OAV), which were then used to evaluate the contribution of each volatile compound to the aromatic profile of the mead.

### 2.6. Organic Acids, Glucides and Alcoholic Compounds by HPLC-RID

The organic acids, glucides, and alcoholic compounds characteristics were performed using HPLC-DAD ESI+. The samples were filtered through the Chromafil Xtra nylon filter of 0.45 µm and 20 μL and further injected into the HPLC system. Agilent 1200 HPLC system equipped with a quaternary pump, solvent degasser, and manual injector coupled with a refractive index detector (RID) (Agilent Technologies, Santa Clara, CA, USA) was used for the separation and quantification of carbohydrates, organic acids, alcohols. Compound separation was performed on a Polaris Hi-Plex H column, 300 × 7.7 mm (Agilent Technologies, Santa Clara, CA, USA), using 5 mM H_2_SO_4_ mobile phase at a flow rate of 0.6 mL/min, column temperature T = 800 °C, and RID temperature T = 35 °C. Elution of the compounds was performed for 25 min. Data acquisition and interpretation of the results were performed using OpenLab-ChemStation C.01.09[144] software (Agilent Technologies, Santa Clara, CA, USA). The identification of compounds in the analyzed samples was conducted by comparing the retention times with those of standard compounds. Data acquisition and interpretation of results were conducted using Agilent ChemStation B.02.01-SR2[260] software. All experiments were conducted in triplicate and the reported data are mean values.

### 2.7. Amino Acids Compounds by Gas-Chromatography

The experiments were analyzed by gas chromatography using Phenomenex EZ:Faast™ kit based the procedure previously followed by [37] with some modifications. The amino acids were extracted from 100 µL of sample and norvaline was used as the internal standard. The kit contains 2 vials of standard AA solutions, a column, reagents, and materials necessary for solid phase extraction and derivatization. Standard solution 1 a mixture of 23 AA: AAA (α-aminoadipic acid), ABA (α-aminobutyric acid), Ala (alanine), Aile (allo-isoleucine), Asp (aspartic acid), βAIB (β-aminoisobutyric acid), C-C (cystine-cystine), Glu (glutamic acid), Gly (glycine), His (histidine), Hyp (4-hydroxyproline), Ile (isoleucine), Leu (leucine), Lys (lysine), Met (methionine), Orn (ornitine), Phe (phenylalanine), Pro (proline), Sar (sarcosine), Ser (serine), Thr (threonine), Tyr (tyrosine), and Val (valine). Standard solution 2 contains the mixture of unstable in acid solution AA: Asn (asparagine), Gln (glutamine), and Trp (tryptophan). Calibration was made for all the amino acids, and the correlation coefficients of all calibration curves were higher than 0.993. An Agilent 6890 N with a flame ionisation detector (FID) was used for the analytical investigation. The column was a Zebron ZBAAA 10 m × 0.25 mm capillary GC column. The column oven temperature programme was: 30 °C/min from 110° to 320 °C. The FID detector temperature was 320 °C and 2.5 µL was injected at an injection temperature of 250 °C and a split level of 1:15. The carrier gas was He at a pressure of 8 psi. Each sample was analyzed in triplicate, the reported data are mean values. Data handling and processing were performed using Empower 2 software.

### 2.8. Statistical Analysis

Data were reported as average mean ± standard deviation (SD) for triplicate determinations. The ANOVA analysis of variance was used to compare the average mean values, using SPSS 19.0 for statistical analysis (IBM, New York, NY, USA) and Tukey’s honestly significant difference (HSD) test with a confidence interval of 95% or 99%. A *p*-value below 0.05 was considered statistically significant.

## 3. Results and Discussion

### 3.1. Total Phenolic Content and Antioxidant Activity

The total phenolic content of each mead sample was found to be significantly different, with values ranging from 166.25 to 194.29 mg GAE/100 mL. These results are consistent with a previous study on Polish mead enriched with rowanberry juice (215.1 mg GAE/100 mL) and root spices (15.27 mg GAE/100 mL) [33]. The mead sample that exhibited the highest concentration of phenolic compounds was V2, with a value of 194.29 mg GAE/100 mL. The V1 mead sample also displayed a relatively high concentration of phenolic compounds, with a value of 191.90 mg GAE/100 mL. However, it is important to note that the co-fermented mead samples, V4 and V3, exhibited lower concentrations of phenolic compounds, with values of 176.90 and 166.25 mg GAE/100 mL, respectively. The results obtained from the co-fermentation experiments are inconsistent with the outcomes reported in previous studies conducted on different matrices, such as tangerine wines [38] and grape wine [39].

The antioxidant activity of the four types of mead showed insignificant deviations, with values ranging from 817.06 to 872.62 mg TE/mL. Notably, the meads subjected to co-fermentation, namely V4 and V3, exhibited the highest antioxidant activity, measuring 872.62 and 844.80 mg TE/mL, respectively. Conversely, the meads fermented with only one yeast strain, V1 and V2, displayed slightly lower antioxidant activity of 833.16 and 817.06 mg TE/mL.

### 3.2. Individual Phenolic Compounds Analysis

The analysis of individual phenolic compounds analysis revealed that the total concentration of phenolic compounds ranged from 257.65 to 401.38 μg/mL for the single culture fermentation experiments (Table 1). Notably, meads produced with *Saccharomyces* strains (V1 and V2) exhibited a higher concentration of phenolic compounds, ranging from 358.74 to 358.74 μg/mL. In contrast, the mixed culture co-inoculations (*T. delbrueckii* and *Saccharomyces*) resulted in lower phenolic compound contents, with a range of 257.65 to 315.44 μg/mL.

The phenolic composition of mead samples showed a notable correlation with the types of yeast used. Meads fermented with *Saccharomyces* strains exhibited higher concentrations of both total phenolic content and individual phenolic compounds, whereas co-fermented meads with mixed cultures demonstrated lower phenolic compound contents. These findings emphasize the significant influence of yeast selection on the phenolic characteristics of mead samples.

Catechin is a flavonoid commonly found in rosehips, with reported concentrations of up to 522.48 µg/g [40]. The mead samples subjected to mono-strain fermentation exhibited higher concentrations of catechin, ranging from 60.72 to 84.22 µg/mL. In contrast, mead samples subjected to sequential fermentation have been associated with lower concentrations of catechin (39.62–48.87 µg/mL). Enzymatic activity produced by microorganisms during mead fermentation leads to a decrease in the concentration of catechin [41], except for V1, which exhibited a higher content of catechin compared to the wort with 5%. One possible reason for this could be a coupled oxidation mechanism involving caffeoylquinic acid, as suggested by Tanaka et al. [42] in their study on fermented tea obtained from loquat and green tea leaves. The study indicates that the oxidation activity of loquat leaf enzymes resulted in the accelerated oxidation of catechin. This acceleration was attributed to a coupled oxidation mechanism with caffeoylquinic acids, the major phenolic constituents of loquat leaves. Drawing parallels to the current study, it is plausible that a similar coupled oxidation mechanism involving caffeoylquinic acid or other phenolic compounds in the mead samples contributed to the higher catechin content in V1. However, further investigations are needed to confirm this hypothesis and understand the specific mechanisms involved.

In terms of phenolic acid content, hydroxybenzoic acids, including 2,3-dihydroxybenzoic acid, gentisic acid, and protocatechuic acid, as well as gallic acid, vanillic acid, and p-coumaric acid were found to be the most significant in the fermented beverages. Gallic, p-coumaric, vanillic, and protocatechuic acids are commonly found in honey and mead and might be responsible for the antioxidant activity of these products [43]. Among them, gallic and gentisic acids were present in the highest amounts, ranging from 13.95 to 22.66 µg/mL and 4.52 to 35.54 µg/mL, respectively. The observed increase in their concentrations after fermentation, up to 9-fold, could be attributed to the fact that hydroxybenzoic acids and p-coumaric acid are abundant in rosehips, with high concentrations of gallic acid (12.93 mg/g dry weight) and p-coumaric acid (6.28 mg/g dry weight) as being previously reported [44]. Interestingly, the co-inoculation of mixed cultures led to a significant decrease in the quantities of gallic and gentisic acids, with reductions ranging from 10 to 19 µg/mL.

Protocatechuic aldehyde, a phenolic compound ubiquitous in various plant and fruit sources, serves as a precursor of protocatechuic acid [45]. Rosehips have been reported to contain protocatechuic acid at concentrations of up to 10 µg/g [46]. During the fermentation process, the concentration of protocatechuic acid decreased and ranged in meads from 9.07 to 16.96 µg/mL. Notably, the content of protocatechuic acid in mead fermented with rosehips was significantly higher than in Polish mead (e.g., 1.14 µg/g) [47], possibly due to the addition of rosehips in meads.

The presence of vanillic acid, a type of hydroxybenzoic acid, was detected in small quantities ranging from 2.47 to 5.84 µg/mL. Following fermentation, the quantity of vanillic acid increased up to 12 times, with the highest amount observed in single strain fermentation V1 and V2 at 5.84 and 4.86 µg/mL respectively, followed by co-fermentation V3 and V4 with quantities ranging from 2.47 to 4.32 µg/mL. Our findings reveal a significantly higher concentration of vanillic acid compared to that reported in Czech mead, which contained only 0.66 µg/g [48].

### 3.3. Volatile Compounds Analysis

A total of 29 compounds were identified and their levels were found to vary significantly depending on the type of yeast used during fermentation (Table 2). The sample with the highest content of volatile compounds was V1, which underwent single-strain fermentation using *S. bayanus*, with a level of 2742.21 μg/mL. The co-fermentation of *T. delbrueckii* in V3 and V4 also resulted in relatively high levels of volatile compounds, with 2285.12 μg/mL and 1907.16 μg/mL, respectively. In contrast, the lowest level of volatile compounds was detected in the V2 sample mead, with a content of only 546.12 μg/mL.

The analysis of the volatile compounds in the four experimental variants, subjected to single-strain fermentation and co-fermentation, revealed a diverse range of volatile compounds such as fatty acids (e.g., hexanoic acid, octanoic acid), alkanes (e.g., cyclopropane. 1.2-dimethyl-. cis-), alcohols (e.g., 1-Butanol, 3-methyl, hexanoic acid, octanoic acid, phenylethyl alcohol), ketones (e.g., 4-oxo-.beta.-isodamascol), and glycosides (e.g., methyl 4-O-methyl-d-arabinopyranoside). The analysis revealed the presence of minor compounds, such as alcohols (e.g., 1-propanol, 3-ethoxy-) and carboxylic acids (e.g., 2-methylpropanoic acid, 2-methylbutanoic acid), as well as lower amounts of 1,2-cyclohexanediol and 1-methyl-4-(1-methylethenyl)-.

Alcoholic compounds are the primary group of volatile compounds found in mead, and they significantly influence the beverage’s sensory properties. The most abundant alcoholic compounds presented in the samples include 1-butanol, 3-methyl; phenylethyl alcohol, 1.2-dimethyl, 2.3-butanediol, and isobutanol similar results have been exhibited in Polish meads [47]. Among these compounds, the concentration of 1-butanol, 3-methyl- in single-strain fermented mead V1 was the highest (1346.98 μg/mL). In contrast, the concentration was significantly lower in V2 (174.23 μg/mL). In the case of mixed-fermented mead, the concentration of 1-butanol, 3-methyl was lower and more constant, with concentrations of 879.18 μg/mL and 1156.36 μg/mL for V3 and V4, respectively, which aligns with previous research [49]. Phenylethyl alcohol was identified as one of the major compounds present in substantial concentration in the mead samples. Similar to 1-butanol, 3-methyl, it exhibited a consistent concentration trend in the co-fermented samples V3 and V4, with concentrations of 823.49 μg/mL and 983.33 μg/mL, respectively. In contrast, single-strain fermentation (V1) resulted in a five-fold increase in the concentration of phenylethyl alcohol compared to V2. Phenylethyl alcohol was associated with multiflora honey-derived mead, according to a study by [45]. However, other research, such as the study conducted by Li et al. [50], has linked phenylethyl alcohol to the presence of yeasty and green aromas in mead.

Higher alcohols are volatile compounds derived from the fermentation process, characterized by their molecular weight and boiling point higher than that of ethanol. These compounds can be classified into three distinct categories based on their amino acid origins: branched-chain, sulphur-containing, and aromatic alcohol. At lower concentrations, higher alcohols contribute positively to the overall complexity of the wine [51]. In the presented study the following higher alcohols were revealed the presence of 1-Propanol (2-methyl-), 1-Butanol (3-methyl-), 1-Propanol (3-ethoxy-), Phenylethyl alcohol, 1-Hexanol (2-ethyl-), and 1-Propanol (3-(methylthio)-).

Furthermore, *Saccharomyces* species display exceptional proficiency in the production of higher alcohols [52]. Additionally, non-*Saccharomyces* wine yeast species, such as *T. delbrueckii*, have gained increasing attention in oenology. While these non-*Saccharomyces* species generally exhibit lower synthesis of aromatic higher alcohols compared to *S. cerevisiae*, their co-inoculation with *S. cerevisiae* has resulted in wines with comparable or even higher levels of volatiles compared to those obtained with *S. cerevisiae* alone [22]. Importantly, our results indicate minimal differences in the quantities of high alcohols, specifically the higher alcohols previously mentioned, between single-strain fermentation and mixed fermentation.

In terms of acetic acid concentration, the V1 mead exhibited the highest acid content (4.87 μg/mL), followed by V4 (4.38 μg/mL), while no acetic acid was detected in V2 and V3 samples. [50] reported that a substantial amount of acetic acid is formed during the mead fermentation process. This acidic compound has been found to have adverse effects on the fermentation process by reducing pH, increasing total acidity, and decreasing the dissociation of fatty acids. High concentrations of acetic acid and fatty acids can cause a significant slowdown in the fermentation process.

Esters such as ethyl acetate and other ethyl esters were found to exhibit varying concentrations depending on the types of yeast used during the fermentation process. These compounds are formed during fermentation by yeasts and contribute in low concentrations to the fruity and floral aroma of mead [53]. Sequential fermented meads showed significantly higher concentrations of ethyl acetate. Pereira et al. [54] reported that ethyl acetate may also contribute to off-flavors specific to Saccharomyces yeast. Furthermore, [12] characterized ethyl acetate in mead with pollen addition noting its detectable solvent-like taste at a concentration of 12 μg/L.f

Fatty acids, including hexanoic acid and octanoic acid, have been associated with malodorous notes such as rancidity and cheesy odors, typically observed at a total concentration of approximately 5004 μg L^−1^ as reported by Carpena et al. [55]. The concentrations of octanoic and hexanoic acid were found to be relatively low in the co-fermentation meads V3 and V4, while V1, which underwent single-strain fermentation, exhibited the highest concentrations. In contrast, V2 had the lowest levels of these fatty acids among the four analyzed meads.

### 3.4. Odour Activity Values

The odor activity values (OAVs) were determined in order to evaluate the potential contribution of volatile compounds to the mead aroma [32]. Thereby, we selected only the most abundant volatile compounds. Only those compounds with an OAV > 1 significantly contribute to the aroma of beverages. However, according to recent research, compounds with an OAV > 0.2 provide some subtle nuances influencing the overall aroma due to the cumulative influence of compounds with similar aroma descriptors [56]. Thus, from the thirty volatile compounds quantified, only nine were above their perception threshold (Table 3).

The highest OAV was attributed to co-fermented mead samples, mostly due to the hexanoic acid.ethyl ester that confers a fruity, aniseed, green apple flavor [32], and ethyl acetate, is recognized as one of the major esters present in alcoholic beverages [36].

The alcohols, 3-methyl-1-butanol, 2-phenylethanol, and 1-propanol.3-ethoxy were present above their odor threshold, particularly in V1 mead and in the co-fermented variants, conferring malty and fruity notes, respectively [15,33,34]. Such as the above-mentioned alcohols, phenylethanol is a positive contributor to mead aroma, being characterized by pleasant roses, flowery, and lilac odor descriptors [12,32].

Two volatile fatty acids, hexanoic and octanoic acids, previously reported with unpleasant aromas of cheesy, sweaty, fatty, rancid or buttery aromas were found, except V2 sample, in all mead above their odor perception threshold, and with similar OAVs. Still, co-fermentation contributed to a lower OAV on these fatty acids.

### 3.5. Determination of Amino Acids Composition

The amino acid (AA) profile of meads consisted of 11 amino acids (Table 4). The co-fermentation of mead with *T. delbrueckii* exhibited the highest total amino acid concentration at 8.33 mg/100 mL, while the single-strain fermentation with S. bayanus showed a significantly lower concentration of 3.31 mg/100 mL. This is consistent with previous research by Chen et al. [57] who found that Saccharomyces cultures produced more amino acids in lychee wines compared to other yeasts. The main amino acids identified in the mead samples were proline, alanine, valine, β-aminoisobutyric acid (BAIBA), asparagine (Asn), and aspartic acid (Asp), with none exceeding 6 mg/100 mL. These findings support previous work [58], which reported that the composition of amino acids affects the formation of volatile compounds that contribute to the aroma of fermented beverages.

Moreover, Yan et al. [59], emphasized that amino acid composition and prevalence play a paramount role in understanding flavor and taste formation, as they act as crucial precursors for synthesizing volatile compounds that contribute to aroma and taste perception. Based on their taste characteristics, amino acids can be classified as follows: sweet amino acids including glycine (Gly), serine (Ser), alanine (Ala), proline (Pro), threonine (Thr), cysteine (Cys), and methionine (Met); bitter amino acids such as arginine (Arg), histidine (His), isoleucine (Ile), leucine (Leu), phenylalanine (Phe), lysine (Lys), tyrosine (Tyr), and valine (Val); and umami amino acids namely aspartic acid (Asp) and glutamic acid (Glu) [60,61].

Following the fermentation process, the results indicate a decrease in the concentration of AA from an initial value of 12.45 mg/100 mL in the wort to a range of 1.95 to 8.33 mg/100 mL. This decline can be attributed to the utilization of amino acids as the primary nitrogen source, which supports the yeast’s normal growth and facilitates the progression of alcoholic fermentation [62]. According to the data presented in Table 3, it is evident that Saccharomyces yeast (V1 and V2) exhibited complete utilization of Ala, BAIBA, Asp, Phe, Lys, His, and Tyr. However, the content of Pro decreased by approximately 50% compared to its initial concentration in the wort. In contrast, when considering mixed fermentation, the content of various amino acids such as Val, BAIBA, Pro, Asp, Phe, Lys, His, and Tyr in V4 remained relatively unchanged.

Proline serves as a major marker of mead quality and is the dominant amino acid in the mead samples, with concentrations ranging from 0.44 to 5.79 mg/100 mL, predominantly found in co-fermented samples, a fact also confirmed by previous findings [63]. In contrast, asparagine, with a quantity ranging between 0.56 to 1.13 mg/100 mL, was primarily found in single-strain fermented mead. It is worth noting that the European Parliament and the Council [64] and have set a minimum proline concentration of 180 mg/kg in honey, with a minimum quantity of 0.05 mg/mL in mead. Our results comply with this regulation and are consistent with the proline content of Czech meads studied by [9,63] (Klikarova, Ceslova, and Fischer 2021). Proline is known to act as an intermediate in the biosynthesis of glutamic acid, as evidenced by the study conducted by [63]. In this study, a direct association was established between proline intake and the residual glutamic acid content in the mead samples. Notably, V2, characterized by the highest proline intake and displayed a significantly higher concentration of glutamic acid at 0.26 mg/100 mL. Moreover, the same sample exhibited the lowest proline concentration, measuring 0.44 mg/100 mL.

Certain amino acids, such as Val, Leu, isoleucine (Ile), Thr, and Phe, can undergo catabolic degradation, resulting in the production of volatile compounds. Specifically, Leu serves as the precursor for isoamyl alcohol (3-methyl-1-butanol), while Val acts as the precursor for isobutyl alcohol (2-methyl-1-propanol), and Ile is the precursor for active amyl alcohol (2-methyl-1-butanol) [65]. Specifically, higher alcohols are synthesized from keto acids derived from their respective amino acids [66]. The degradation of amino acids occurs through two pathways: Ehrlich pathway and the biosynthesis route using the carbon source. In the Ehrlich pathway, amino acids are converted into keto acids through decarboxylation reactions [67]. Subsequently, these keto acids undergo further enzymatic transformations to yield higher alcohols as end products. On the other hand, the biosynthesis route involves the direct conversion of amino acids into higher alcohols through specific enzymatic reactions, utilizing the available carbon source during fermentation [68].

The analysis of the volatile and amino acids data revealed a notable correlation between the concentrations of valine and the production of 1-butanol. Specifically, higher levels of valine (V1 and V3) coincide with increased production of 1-butanol (V1), whereas lower concentrations of valine (V2 and V4) align with decreased production of 1-butanol (V2). This correlation suggests a potential influence of valine on the synthesis of 1-butanol, which is a derivative of isobutanol. Considering the inherent capacity of S. cerevisiae to produce higher alcohols, it emerges as a promising candidate for the industrial production of isobutanol according to [69].

Hazelwood et al. [70] observed a direct link between the concentration of phenylalanine and the production of phenylethyl alcohol. Phenylalanine, metabolized through the Ehrlich pathway, serves as a precursor for the synthesis of fusel acids, including phenylethyl alcohol, which suggests its involvement in the export of fusel acids. Our data indicate a positive correlation between the concentration of phenylalanine and the production of phenylethyl alcohol. This correlation is supported by the higher concentration of phenylethyl alcohol observed in V1, V3, and V4, where the phenylalanine concentration is either non-detectable (nd) or relatively low amounts.

### 3.6. Determination of Sugars, Organic Acids and Ethanol

In terms of the predominant sugars in mead samples during the fermentation process, glucose and fructose were identified as the major sugars, with the highest concentrations measured at the wort stage at 52.19 and 58.19 mg/mL, respectively (Table 5). However, the concentrations of both sugars decreased significantly after fermentation, dropping below 2 mg/mL. Regarding the effect of single-strain fermentation versus co-fermentation on glucose and fructose concentrations in the mead samples, the results showed that single-strain fermentation led to higher concentrations of glucose compared to co-fermentation. Specifically, glucose concentration was measured at 1.25 and 1.68 mg/mL for V1 and V2, respectively. Whereas co-fermentation with *T. delbrueckii* resulted in glucose concentrations ranging from 0.71 to 1.22 mg/mL, owing to the moderate fermentative capacity of the non-*Saccharomyces* yeast [22]. The fructose concentrations exhibited a similar trend across the samples, in agreement with other studies [71,72].

According to [73], lower levels of glucose can hinder the formation of higher alcohols due to the reduced yeast cell density observed under such conditions. In our study, we confirmed that the concentration of glucose was significantly higher in the Wort sample compared to the other samples (V1, V2, V3, and V4). This higher glucose concentration in the wort likely led to a higher yeast cell density, which could have facilitated the production of higher alcohols. To investigate the relationship between glucose levels and specific higher alcohols (1-Propanol. 2-methyl-, 1-Butanol. 3-methyl-, Phenylethyl Alcohol, and 1-Hexanol, 2-ethyl-), we examined their concentrations in different samples and their correlation with glucose levels. Significantly higher levels of 1-Propanol. 2-methyl- (V1 = 21.72 μg/mL, V4 = 22.55 μg/mL) and Phenylethyl Alcohol (V1 = 1075.74 μg/mL, V4 = 983.33 μg/mL) were observed in the V1 and V4 samples, which had higher glucose concentrations compared to the V2 and V3 samples. This suggests that the elevated glucose levels likely facilitated a higher yeast cell density, thereby promoting the synthesis of these higher alcohols. Conversely, the V2 and V3 samples with lower glucose concentrations showed significantly lower levels of 1-Butanol,3-methyl- (V2 = 174.23 μg/mL, V3 = 879.18 μg/mL) compared to the V1 and V4 samples. This decrease in 1-Butanol,3-methyl- levels could be attributed to the inhibition of higher alcohol formation resulting from the lower glucose levels and the associated lower yeast cell density.

Ethanol is a primary product of the fermentation process, and its concentration in the mead samples varies from 20.58 to 45.59 mg/mL. The use of *S. cerevisiae* yeast in the fermentation process produced significantly higher alcohol concentrations of 45.59 and 35.01 mg/mL, compared to mixed fermentation, in accordance with the residual sugar content.

Sequential fermentation with *T. delbrueckii* yeast (V3 and V4) resulted in lower ethanol production, with levels ranging between 20.58 and 32.77 mg/mL. This finding is in line with previous studies by [22,74] which reported reduced ethanol and volatile acidity production in beverages fermented with *T. delbrueckii*. The observations are consistent with the metabolic profile of *T. delbrueckii* as a non-*Saccharomyces* yeast, known for its tendency to exhibit lower ethanol production compared to typical Saccharomyces yeast strains. Notably, in the mead sample that was inoculated with *T. delbrueckii* and co-fermented with *S. cerevisiae*, moderate concentrations of glucose, fructose, and ethanol were observed. Specifically, the concentrations were measured at 1.22 mg/mL for glucose, 0.81 mg/mL for fructose, and 32.77 mg/mL for ethanol. This suggests that the presence of *T. delbrueckii* in the co-fermentation process influenced the levels of these compounds in the final product.

The main organic acids in the mead samples were citric, malic, lactic, and acetic acid (Table 5). The content of organic acids in the mead samples varied greatly, ranging from 0.25 to 3.17 mg/mL, with lactic acid being the most abundant, consistent with previous studies, although its concentration may vary depending on the floral or geographical origin of the honey [75].

Succinic acid, a metabolite synthesized during the fermentation process, was not detected in the initial wort, as expected. However, subsequent analysis of the mead samples revealed the presence of succinic acid with concentrations ranging from 0.58 to 0.85 mg/mL. It is noteworthy that there were no statistically significant differences in succinic acid levels observed between the single strain and co-fermentation procedures, validating the findings reported by [76]. Specifically, the strain *S. cerevisiae* (sample V1) exhibited the highest concentration of succinic acid at 0.85 mg/mL, while V4 displayed the lowest concentration at 0.58 mg/mL, consistent with the findings reported in the study conducted by [32].

Acetic acid concentration increased after fermentation, with low concentrations observed only in the co-fermented mead, with quantities of 0.35 and 0.16 mg/mL, respectively. The concentration of acetate depends mainly on the concentration of carbohydrates and the source of nitrogen. Therefore, we can assume that *T. delbrueckii* demonstrated superior utilization of the available nutrients, including glucose and nitrogen [77], compared to single-strain *S. cerevisiae* fermented mead [78].

The analysis of citric acid content in the mead samples revealed a significant post-fermentation increase, ranging from 1.01 to 2.20 mg/mL. Notably, the single-strain fermentations exhibited the highest concentration of citric acid, with values of 2.20 and 1.91 mg/mL, respectively, while the co-fermented samples exhibited approximately half of these concentrations. High levels of citric acid in mead might have a negative impact on its aroma by imparting a vinegar taste [15,79].

## 4. Conclusions

Our study depicts the impact of yeast selection and fermentation techniques on the chemical composition of rosehip mead. Mono-strain fermentation utilizing *Saccharomyces cerevisiae* demonstrated the preservation of rosehip-specific flavonoids, particularly catechin, and phenolic acids such as protocatechuic acid. Furthermore, *Saccharomyces bayanus* exhibited elevated concentrations of volatile compounds, including 1-butanol and 3-methyl, as well as the synthesis of higher alcohols such as Propanol (2-methyl-), 1-Butanol (3-methyl-), 1-Propanol (3-ethoxy-), Phenylethyl alcohol, 1-Hexanol (2-ethyl-), and 1-Propanol (3-(methylthio)-). This yeast strain also contributed to the presence of fatty acids associated with malodorous notes, highlighting the significant influence of *Saccharomyces* yeast on the overall chemical profile, glucose levels, and alcohol production in rosehip mead.

Conversely, sequential fermentation involving non-*Saccharomyces* yeast, specifically *Torulaspora delbrueckii*, led to diminished concentrations of select phenolic acids, such as gallic and gentisic acids. However, it demonstrated notable proficiency in ester generation, particularly ethyl acetate, which imparts fruity and floral aromatic nuances to the mead. Notably, mixed fermentation resulted in sustained levels of essential amino acids, including valine, β-aminoisobutyric acid, proline, aspartic acid, phenylalanine, lysine, histidine, and tyrosine. A correlation emerged between valine and the biosynthesis of 1-butanol, a derivative of isobutanol, while the concentration of phenylalanine displayed discernible associations with the production of specific volatile compounds.

## Figures and Tables

**Table 1:** Individual phenolic compounds expressed in μg/mL.

Compunds	Wort	V1	V2	V3	V4	Sig
p-Anisaldehyde	9.36 ± 2.07 ^ab^	12.21 ± 1.52 ^a^	8.81± 1.91 ^ab^	5.74 ± 0.73 ^ab^	8.38 ± 1.47 ^ab^	*
Gallic acid-glucoside	15.08 ± 1.03 ^d^	41.47 ± 4.72 ^a^	35.79 ± 2.19 ^ab^	22.83 ± 1.01 ^c^	31.89 ± 2.02 ^b^	***
2,3-Dihydroxybenzoic acid	13.65 ± 2.07 ^c^	25.76 ± 0.90 ^a^	23.67 ± 1.04 ^a^	18.84 ± 0.96 ^b^	23.69 ± 1.04 ^a^	**
Gallic acid	13.95 ± 1.24 ^c^	22.66 ± 1.56 ^a^	21.24 ± 1.94 ^a^	16.57 ± 0.96 ^bc^	19.90 ± 0.86 ^ab^	**
Ethyl gallate	15.63 ± 0.99 ^b^	33.43 ± 1.04 ^a^	38.22 ± 3.00 ^a^	34.44 ± 1.24 ^a^	33.65 ± 2.03 ^a^	*
Gentisic acid	4.52 ± 0.30 ^d^	35.55±2.33 ^a^	30.70 ± 2.94 ^ab^	22.73 ± 1.05 ^c^	28.05 ± 1.76 ^b^	***
Protocatechuic aldehyde	8.64 ± 1.25 ^d^	82.81 ± 3.02 ^a^	81.81 ± 2.15 ^a^	53.71 ± 1.92 ^c^	66.44 ± 2.36 ^b^	***
Protocatechuic acid	25.74 ± 1.43 ^a^	16.96 ± 0.26 ^b^	17.80 ± 1.45 ^b^	9.07 ± 0.84 ^c^	10.12 ± 0.92 ^c^	***
3-Caffeoyquinic acid	3.71 ± 0.61 ^d^	10.59 ± 0.75 ^bc^	9.78 ± 0.70 ^c^	12.80 ± 1.02 ^b^	15.69 ± 1.11 ^a^	***
Catechin	80.18 ± 0.46 ^a^	84.22 ± 0.81 ^a^	60.72 ± 5.84 ^b^	39.62 ± 0.37 ^d^	48.87 ± 0.28 ^c^	***
5-Caffeoyquinic acid	3.35 ± 0.24 ^d^	19.64 ± 1.14 ^a^	16.96 ± 1.32 ^b^	13.38 ± 0.32 ^c^	16.22 ± 1.12 ^b^	***
Vanilic acid	0.46 ± 0.06 ^d^	5.84 ± 1.06 ^a^	4.86 ± 1.35 ^a^	2.47 ± 0.24 ^bc^	4.32 ± 0.60 ^ab^	***
p-Coumaric acid	3.49 ± 0.34 ^d^	10.18 ± 2.30 ^a^	8.33 ± 0.76 ^ab^	5.51 ± 1.65 ^bc^	8.18 ± 0.47 ^ab^	***
Total Phenolics	197.77 ± 11.72 ^d^	401.34 ± 19.35 ^a^	358.70 ± 21.94 ^b^	255.89 ± 12.00 ^c^	315.40 ± 15.28 ^b^	***

Values are expressed as mean of three replicates. Values with different lower letters in the same row indicate statistically significant differences between samples (Tukey’s test, *p* < 0.05). * significant *p* ≤ 0.05; ** very significant *p* ≤ 0.01; *** extremely significant *p* ≤ 0.001.

**Table 2:** Volatile compounds expressed in μg/mL.

Compounds	V1	V2	V3	V4	Sig
Cyclopropane. 1.2-dimethyl-. cis-	23.47 ± 0.26 ^c^	114.63 ± 1.06 ^a^	90.87 ± 0.85 ^b^	11.16 ± 0.10 ^d^	***
1-Propanol. 2-methyl-	21.72 ± 0.19 ^a^	nd	15.97 ± 0.15 ^b^	22.55 ± 0.21 ^a^	**
1-Butanol. 3-methyl-	1346.98 ± 13.48 ^a^	174.23 ± 1.65 ^d^	879.18 ± 9.33 ^c^	1156.36 ± 10.42 ^b^	***
Hexanoic acid. ethyl ester	3.19 ± 0.03	nd	nd	nd	
Propanoic acid. 2-hydroxy-. ethyl ester	6.83 ± 0.06 ^a^	nd	3.11 ± 0.02 ^b^	3.99 ± 0.04 ^b^	**
1-Propanol. 3-ethoxy-	12.14 ± 0.13 ^b^	3.02 ± 0.03 ^c^	11.96 ± 0.11 ^b^	13.36 ± 0.46 ^a^	***
Acetic acid	4.87 ± 0.04 ^a^	nd	nd	4.38 ± 0.04 ^a^	ns
2.3-Butanediol. [R-(R@.R@)]-	3.79 ± 0.03	nd	nd	nd	
Propanoic acid. 2-methyl-	5.44 ± 0.05 ^c^	6.64 ± 0.06 ^b^	nd	12.92 ± 0.11 ^a^	***
Butanoic acid	3.11 ± 0.04	nd	nd	nd	
Butanedioic acid. diethyl ester	3.33 ± 0.03	nd	nd	nd	
Butanoic acid. 2-methyl-	9.52 ± 0.09 ^a^	nd	3.22 ± 0.03 ^c^	5.24 ± 0.05 ^b^	***
1.3-Propanediol. diacetate	15.04 ± 0.17	nd	nd	nd	
Methyl 4-O-methyl-d-arabinopyranoside	32.54 ± 0.29 ^a^	nd	11.45 ± 0.11 ^b^	10.10 ± 0.09 ^c^	***
Hexanoic acid	32.96 ± 0.30 ^a^	nd	6.53 ± 0.07 ^b^	4.82 ± 0.05 ^c^	***
Phenylethyl Alcohol	1075.74 ± 9.56 ^a^	221.71 ± 2.04 ^d^	823.49 ± 8.91 ^c^	983.33 ± 8.72 ^b^	***
Octanoic Acid	27.58 ± 0.25 ^a^	4.22 ± 0.05 ^d^	9.92 ± 0.09 ^b^	8.70 ± 0.08 ^c^	***
1,2-Cyclohexanediol, 1-methyl-4-(1-methylethenyl)-	6.69 ± 0.07 ^a^	1.33 ± 0.02 ^c^	3.05 ± 0.04 ^b^	3.16 ± 0.03 ^b^	***
Ethyl hydrogen succinate	25.78 ± 0.23	nd	nd	nd	
4-Oxo-.beta.-isodamascol	81.48 ± 0.82 ^a^	16.74 ± 0.16 ^c^	23.24 ± 0.24 ^b^	27.03 ± 0.27 ^b^	***
Ethyl Acetate	nd	3.12 ± 0.02 ^c^	4.42 ± 0.05 ^b^	5.42 ± 0.05 ^a^	***
Butanoic acid, 3-methyl-, ethyl ester	nd	5.42 ± 0.05	nd	nd	
1-Propanol, 3-(methylthio)-	nd	nd	4.77 ± 0.07	nd	
2H-Pyran-2,6(3H)-dione	nd	nd	1.21 ± 0.02 ^b^	2.57 ± 0.03 ^a^	***
1-{2-[3-(2-Acetyloxiran-2-yl)-1, 1-dimethylpropyl]cycloprop-2-enyl}ethanone	nd	nd	3.54 ± 0.03	nd	
Benzenemethanol, 3,4-dimethoxy-	nd	nd	4.60 ± 0.04	nd	
2-Butanone, 3-hydroxy-	nd	nd	nd	1.53 ± 0.01	
1-Hexanol, 2-ethyl-	nd	nd	nd	1.07 ± 0.01	
1-Propanol, 3-(methylthio)-	nd	nd	nd	4.70 ± 0.04	
Methyl 4-O-methyl-d-arabinopyranoside	nd	nd	nd	10.10 ± 0.09	

Values are expressed as mean of three replicates. Values with different lower letters in the same row indicate statistically significant differences between samples (Tukey’s test, *p* < 0.05). ** very significant *p* ≤ 0.01; *** extremely significant *p* ≤ 0.001; ns = not significant; nd = not detected.

**Table 3:** Odor activity values (OAV) of volatile compounds of more influence on the aroma of mead.

Compounds	Odor Descriptors ^a^	Odor Threshold (µg/L)	V1	V2	V3	V4	References
1-Propanol. 2-methyl-	Whiskey; malty	5	4.3	nd	3.2	4.5	[33,34]
1-Butanol. 3-methyl-	cheese; nail polish; malty	30,000	44.9	5.8	29.3	38.5	[32,33]
Hexanoic acid. ethyl ester	fruity; aniseed; green apple	14	227.8	nd	nd	nd	[12,32]
1-Propanol. 3-ethoxy-	Fruity	100	121.4	30.2	119.6	133.6	[15]
2,3-Butanediol	Fruity, sweet, butter	150,000	<0.2	nd	nd	nd	[33,35]
Hexanoic acid	cheese; sweaty	420	78.5	nd	15.5	11.5	[32]
Phenylethyl Alcohol	roses; flowery; lilac	14,000	76.8	15.8	58.8	70.2	[12,32]
Octanoic Acid	fatty; rancid; butter; cheese	500	55.2	8.4	19.8	17.4	[12,32]
Ethyl Acetate	solvent; nail polish	12	nd	260	368	452	[12,32]

^a^ Odor descriptors reported in the literature; nd = not detected.

**Table 4:** Amino acids expressed in mg/100 mL.

Compounds	Wort	V1	V2	V3	V4	Sig
alanine (Ala)	0.14 ± 0.02 ^a^	nd	nd	0.15 ± 0.04 ^a^	0.14 ± 0.02 ^a^	ns
valine (Val)	0.01 ± 0.00 ^b^	nd	0.12 ± 0.03 ^a^	nd	0.01 ± 0.00 ^b^	**
b-aminoisobutyric acid (βAIB)	0.19 ± 0.01 ^a^	nd	nd	nd	0.20 ± 0.01 ^a^	ns
proline (Pro)	5.78 ± 0.95 ^a^	2.23 ± 0.09 ^b^	0.44 ± 0.03 ^c^	0.72 ± 0.03 ^c^	5.78 ± 0.95 ^a^	**
asparagine (Asn)	nd	1.08 ± 0.05 ^a^	1.13 ± 0.03 ^a^	0.59 ± 0.28 ^b^	nd	*
aspartic acid (Asp)	0.31 ± 0.02 ^ab^	nd	nd	0.59 ± 0.33 ^a^	0.31 ± 0.01 ^ab^	ns
glutamic acid(Glu)	nd	nd	0.26 ± 0.02	nd	nd	
phenylalanine (Phe)	0.75 ± 0.03 ^a^	nd	nd	nd	0.76 ± 0.03 ^a^	ns
lysine (Lys)	0.33 ± 0.02 ^a^	nd	nd	nd	0.33 ± 0.02 ^a^	ns
histidine (His)	0.46 ± 0.05 ^a^	nd	nd	nd	0.46 ± 0.05 ^a^	ns
tyrosine (Tyr)	0.33 ± 0.04 ^a^	nd	nd	nd	0.33 ± 0.04 ^a^	ns
Total	12.45 ± 1.13 ^a^	3.31 ± 0.14 ^c^	1.95 ± 0.06 ^d^	2.05 ± 0.61 ^d^	8.33 ± 1.13 ^b^	***

Values are expressed as mean of three replicates. Values with different lower letters in the same row indicate statistically significant differences between samples (Tukey’s test, *p* < 0.05). * significant *p* ≤ 0.05; ** very significant *p* ≤ 0.01; *** extremely significant *p* ≤ 0.001; ns = not significant.

**Table 5:** Organic acids, sugars and ethanol expressed in mg/mL.

Compound	Wort	V1	V2	V3	V4	Sig
Oxalic acid	0.29 ± 0.01 ^d^	0.81 ± 0.04 ^a^	0.71 ± 0.07 ^ab^	0.45 ± 0.07 ^c^	0.64 ± 0.03 ^b^	***
Citric acid	0.25 ± 0.03 ^c^	2.20 ± 0.15 ^a^	1.91 ± 0.07 ^a^	1.01 ± 0.07 ^b^	1.72 ± 0.37 ^a^	**
Glucose	52.19 ± 2.08 ^a^	1.68 ± 0.09 ^b^	1.25 ± 0.05 ^b^	0.71 ± 0.03 ^b^	1.22 ± 0.13 ^b^	*
Fructose	58.19 ± 2.26 ^a^	0.94 ± 0.02 ^b^	0.73 ± 0.01 ^b^	0.47 ± 0.02 ^b^	0.81 ± 0.02 ^b^	*
Sorbitol	nd	0.24 ± 0.03 ^a^	0.20 ± 0.04 ^ab^	0.17 ± 0.01 ^b^	0.18 ± 0.01 ^ab^	*
Succinic acid	nd	0.85 ± 0.05 ^a^	0.69 ± 0.02 ^b^	0.58 ± 0.05 ^c^	0.78 ± 0.03 ^ab^	**
Lactic acid	0.67 ± 0.02 ^c^	3.17 ± 0.27 ^a^	2.31 ± 0.02 ^b^	2.05 ± 0.64 ^b^	2.74 ± 0.10 ^ab^	**
Acetic acid	nd	nd	nd	0.35 ± 0.47 ^a^	0.16 ± 0.02 ^a^	ns
Ethanol	4.41 ± 0.08 ^d^	45.64 ± 1.50 ^a^	35.01 ± 0.49 ^b^	20.58 ± 0.10 ^c^	32.77 ± 1.25 ^b^	***

Values are expressed as mean of three replicates. Values with different lower letters in the same row indicate statistically significant differences between samples (Tukey’s test). * significant *p* ≤ 0.05; ** very significant *p* ≤ 0.01; *** extremely significant *p* ≤ 0.001; ns = not significant; nd = not detected.

## Data Availability

Data available on request due to restrictions e.g., privacy or ethical.
